# Effect of Alcohol Swab on Blood Glucose Readings Using a Glucometer: A Quasi-Experimental Study

**DOI:** 10.7759/cureus.98199

**Published:** 2025-11-30

**Authors:** Duaa T Al-aithan, Kawthar Aldandan, Fatima T Aleithan, Feras A Albaqshi, Azhar Al-Ibrahem, Fatema Aldandan

**Affiliations:** 1 Family Medicine, Al-Ahsa Health Cluster, Ministry of Health, Al-Ahsa, SAU; 2 Laboratory, King Fahad Military Medical Complex, Ministry of Defense, Dhahran, SAU; 3 College of Medicine, King Faisal University, Ministry of Education, Al-Ahsa, SAU

**Keywords:** alcohol based hand rub, alcohol products, alcohol swab, alcohol swabbing, capillary blood glucose monitoring, effect, effect of alcohol swab, finger capillary blood glucose, glucometer

## Abstract

Background

Although alcohol swabs are commonly used to disinfect the skin before capillary blood glucose testing, there is inconsistent evidence regarding their actual impact on glucose readings. Some studies have suggested that residual alcohol may lead to falsely elevated results, while others have found no significant effect. This study aimed to evaluate the impact of various finger-cleaning techniques on capillary blood glucose readings obtained using a glucometer.

Methods

A quasi-experimental design was conducted among 249 adult participants attending primary health care centers in Al-Ahsa, Saudi Arabia. The study was conducted between July and September 2025. For each participant, baseline glucose levels were first measured after standard handwashing using capillary sampling. Participants were then divided into three groups: those who washed their hands with water and were tested after their hands had been dried, those whose fingers were disinfected with 70% alcohol and tested while still wet, and those whose fingers were disinfected with 70% alcohol and tested after complete drying. Capillary blood was collected under each condition by trained healthcare providers and analyzed using a calibrated Accu-Chek Instant glucometer (Roche Diagnostics, Germany).

Results

The participants had a median age of 35 years (interquartile range [IQR]: 26-44), and 57% (n = 141) were female. Most participants (90%, n = 225) reported no underlying health conditions. The comparison of blood glucose readings revealed no statistically significant differences between the blood glucose levels measured at baseline after handwashing using capillary sampling and both the hand wash intervention (95, 89.5-103 mg/dL; p = 0.873) and wet-alcohol finger groups (95, 86-107 mg/dL; p = 0.735). However, the dry-alcohol group (98, 91-109 mg/dL) demonstrated higher readings (p = 0.028). Overall, the Kruskal-Wallis test indicated no significant difference in glucose levels among the three intervention groups compared to the baseline level in each participant (p = 0.325).

Conclusions

The study's findings indicate that the blood glucose level is not significantly impacted by cleaning the fingertip with or without an alcohol swab before blood glucose testing, regardless of whether the alcohol is allowed to dry completely. Thus, using alcohol swabs to disinfect hands remains a safe and effective method for both clinical and home glucose testing.

## Introduction

Diabetes mellitus is a metabolic disease affecting millions of individuals worldwide, and the global increase in the prevalence of type 2 diabetes is well documented [1-2]. The member states of the Arab Gulf Cooperation Council, namely Saudi Arabia, Bahrain, Qatar, Oman, Kuwait, and the United Arab Emirates, are among the regions most affected by the global diabetes epidemic [3]. The latest data from the International Diabetes Federation indicate a significant prevalence rate ranging between 8% and 22% across these countries [3]. Blood glucose monitoring is essential in the management of diabetes mellitus [4]. The glucometer, a widely used point-of-care device, allows for quick glucose level assessment via capillary blood samples [5-6]. Some guidelines recommend cleaning the fingertip with an alcohol swab before pricking to prevent infection [7]. However, residual alcohol may contaminate the sample or alter the chemistry of the test strip, leading to inaccurate results [8]. Some suggest using soap and water instead of alcohol swabs, unless soap and water are unavailable, emphasizing that alcohol is not necessary if the skin is cleaned with soap [9].

Literature review

A common practice before obtaining a capillary blood sample is to cleanse the fingertip with an alcohol swab [10]. Some international health organizations consider washing hands with soap and water sufficient for hand disinfection prior to blood sampling, while the use of alcohol swabs is considered an alternative option [5-11]. Multiple studies have investigated whether cleaning the skin with alcohol swabs affects the accuracy of glucometer measurements [12-13].

A study conducted in Iran in 2024 used a randomized quasi-experimental quantitative design involving 160 diabetic patients. Blood glucose levels were measured using a glucometer in three ways: immediately after cleaning the finger with an alcohol swab, 30 seconds after the alcohol had dried, and through venous blood sampling. The results showed no statistically significant differences among the three measurements. The researchers concluded that using alcohol-containing swabs for hand disinfection does not have a significant effect on glucometer readings [12].

In Indonesia (2024), another study aimed to determine the differences in blood glucose levels measured by a glucometer when the finger was still wet with 70% alcohol compared to when it was dry. This study employed a laboratory experimental design and involved 106 capillary blood samples collected from students. The findings indicated a significant difference in glucose readings between specimens obtained from wet and dry fingers. The results suggested that alcohol residue on wet fingers can mix with capillary blood, resulting in lower glucose levels compared to specimens from dry fingers [13].

A study conducted in Jeddah, Saudi Arabia, in 2022 involved family medicine residents at King Saud bin Abdulaziz University. Capillary blood samples were collected from 98 residents to measure glucose levels before and after handwashing or following the use of alcohol swabs and hand sanitizers. The study found no significant differences in glucose levels between samples collected after these hand-cleaning methods and those taken from unwashed hands. However, a significant difference was observed between glucose levels in the first and second drops of blood from both washed and unwashed hands [14].

Notwithstanding the World Health Organization's (WHO) guidance to allow a 30-second drying period after disinfecting a region with a 70% alcohol-soaked swab, it is occasionally noted that nurses or patients at home proceed to pierce the spot immediately following disinfection. This may result in alcohol interfering with glucose monitoring and diluting the blood. Certain literature also advises against discarding the initial drop of blood and using the subsequent drop after cleaning [15]. Stein demonstrated that alcohol swabs alter the blood glucose level measured by a glucometer [16]. This study aims to investigate the effect of alcohol swabs on blood glucose levels using the glucometer.

Study rationale

There is inconsistent evidence regarding the actual impact of alcohol swabbing on glucose readings [12-13]. Moreover, limited evidence is available from the Saudi population concerning the accuracy of capillary blood glucose testing, particularly when using commonly available glucometers such as the Accu-Chek Instant. Previous international studies may have utilized devices with varying sensitivity to residual alcohol, which could have influenced their measurement outcomes [13-14]. Recognizing this gap, the present study was designed to generate local evidence and evaluate whether similar findings apply within the Saudi context.

Objective

The study aimed to compare capillary blood glucose readings obtained under three different finger-cleaning conditions: handwashing with water, alcohol swabbing while the finger was still wet, and alcohol swabbing after complete drying, to determine whether the use of alcohol swabs, either before or after drying, significantly affects the accuracy of glucose readings compared with handwashing. Additionally, the study aimed to provide locally relevant data that could support or refine existing international recommendations.

## Materials and methods

This study aimed to evaluate the differences in blood glucose readings obtained from capillary samples collected under three distinct finger-cleaning conditions: handwashing with water, disinfection with 70% alcohol while still wet, and disinfection with 70% alcohol after complete drying. A quasi-experimental design was employed to investigate the impact of various sampling techniques on the accuracy of glucose measurements using a glucometer.

The study was conducted between July and September 2025, following the approval of the research protocol by the institutional ethics committee on July 21, 2025. The research proposal was prepared and submitted for ethical review between May and July 2025, before the commencement of data collection.

Study population and setting

The study population consisted of patients attending primary health care centers in Al-Ahsa, Saudi Arabia. This population was selected for its accessibility and its diverse physiological characteristics, which were expected to yield representative data that met the study’s inclusion criteria.

Sampling technique

A total of 249 participants were recruited through purposive sampling based on predetermined inclusion and exclusion criteria. For each participant, baseline glucose levels were first measured after standard handwashing using capillary sampling. Then, the participants were systematically allocated into three equal intervention groups, with 83 subjects in each. Group 1 (Handwashing group) samples were obtained from fingers cleaned with water and then dried. This group was created to eliminate the stress of the second sample. Group 2 (Wet-alcohol group), samples were collected within 30 seconds after cleaning with a 70% alcohol swab (before complete drying). Group 3 (Dry-alcohol group), samples were collected after the cleaning with a 70% alcohol swab, and the alcohol had fully evaporated.

Inclusion and exclusion criteria

Eligible participants were adults (≥18 years old) in good general health who voluntarily agreed to participate, had no history of diabetes, and were not taking medications known to affect blood glucose levels. Exclusion criteria included individuals with dermatological conditions affecting all fingers, those with alcohol allergies, or diabetic patients currently using anti-hyperglycemic therapy, as these factors could interfere with glucose readings. These criteria were established to ensure that any observed differences reflected the impact of sampling techniques rather than underlying health conditions.

Materials and instruments

The study utilized an Accu-Chek Instant glucometer (Roche Diagnostics, Germany), glucose test strips, lancets, 70% alcohol swabs, cotton balls, and disposable gloves. We used the Yellow Springs Instruments (YSI) 2300 Stat Plus Glucose and Lactate analyzer (Yellow Springs, OH) as a reference tool to assess the accuracy of the Accu-Chek Instant glucometer. Using a membrane-bound enzyme electrochemical approach in which D-glucose is oxidized in the presence of glucose oxidase, this reference laboratory analyzer measures the amount of glucose in whole blood using an electrochemical probe. Every time data was collected, a fresh membrane was utilized. Initially, 180 mg/dL and 900 mg/dL solutions were used to calibrate the analyzer. Additionally, a 180 mg/dL solution was used for automated quality control, which was carried out in triplicate every 45 minutes. A finger stick was used to draw blood, which was then transferred into two or three heparinized capillary tubes. After that, the blood was combined in a microcentrifuge tube. For duplicate samples using the YSI, two 25 µL samples were successively sucked and evaluated by the analyzer. On the YSI, a single probe was configured to measure whole blood glucose. As a result, we calculated the average of the duplicate samples and reported one value for each sample. According to the International Federation of Clinical Chemistry, all given results are plasma glucose readings, which are adjusted by multiplying the total blood glucose value by 1.11 [17].

Procedure

The procedure began with the preparation of all necessary materials. For each participant, baseline glucose levels were first measured after standard handwashing using capillary sampling. A second glucose level was measured according to the intervention group. Capillary blood was drawn using a sterile lancet and immediately applied to a glucose test strip inserted into a calibrated Accu-Chek Instant glucometer (Roche Diagnostics, Germany). Once the blood was fully absorbed, the glucometer automatically displayed the glucose concentration, which was recorded for analysis. All blood glucose measurements were performed by trained health care providers under the supervision of the principal investigator.

Statistical analysis

Descriptive statistics were used to summarize participants’ demographic and clinical characteristics. Continuous variables were expressed as the median and interquartile range (Q1, Q3), while categorical variables were presented as frequencies and percentages. The Wilcoxon signed-rank test was used to compare differences in blood glucose levels within each intervention group (handwashing, wet-alcohol, and dry-alcohol). Comparisons of glucose levels between the three intervention groups were performed using the Kruskal-Wallis rank-sum test. Statistical significance was set at a p-value < 0.05. All analyses were conducted using Excel and IBM SPSS software (Statistical Package for the Social Sciences; IBM Corp., Armonk, NY, USA) version 26 for Microsoft Windows [14].

## Results

A total of 249 participants were enrolled in the study with no dropouts. More than half of the study cohort consisted of females (57%, n = 141). The median age of the participants was 35 years (interquartile range, IQR: 26-44). The majority of blood glucose measurements (59%) were obtained through random blood sugar samples (n = 147), while fasting blood sugar samples accounted for 41% (n = 102). Most participants (90%, n = 225) reported no underlying morbid conditions (Table 1).

**Table 1:** Demographic and Clinical Characteristics of the Participants ^1^Median (Q1, Q3); n (%)

Characteristic	N = 249^1^
Age	35 (26-44)
Sex	
Female	141 (57%)
Male	108 (43%)
Blood glucose measurement	
Fasting blood sugar	102 (41%)
Random blood sugar	147 (59%)
Disease	
Bronchial Asthma	1 (0.4%)
Epilepsy	1 (0.4%)
Glucose-6-Phosphate Dehydrogenase Deficiency	1 (0.4%)
Hypertension	15 (6.0%)
Hypothyroidism	1 (0.4%)
Irritable bowel syndrome	1 (0.4%)
Iron deficiency	1 (0.4%)
Sickle cell disease	1 (0.4%)
Sickle cell trait	1 (0.4%)
On medication	1 (0.4%)
Free of diseases	225 (90%)

The comparison of blood glucose readings revealed no statistically significant differences between the blood glucose levels measured at baseline after handwashing using capillary sampling and both the hand wash intervention (95, 89.5-103 mg/dL; p = 0.873) and wet-alcohol finger groups (95, 86-107 mg/dL; p = 0.735). However, the dry-alcohol group (98, 91-109 mg/dL) demonstrated significantly higher blood glucose readings (p = 0.028) (Table 2).

**Table 2:** Comparison of Blood Glucose Levels (mg/dL): Intervention vs Baseline Groups ^1^Median (Q1, Q3) ^2^Wilcoxon signed-rank test

Variables	Blood glucose levels (mg/dL) at intervention	Blood glucose levels (mg/dL) at baseline	p-value
Hand wash	95 (89.5-103)	93 (89-107)^1^	0.873^2^
Alcohol wet	95 (86-107)	95 (86-107.5)	0.735
Alcohol dry	98 (91-109)	96 (89-108.5)	0.028

**Figure 1:**
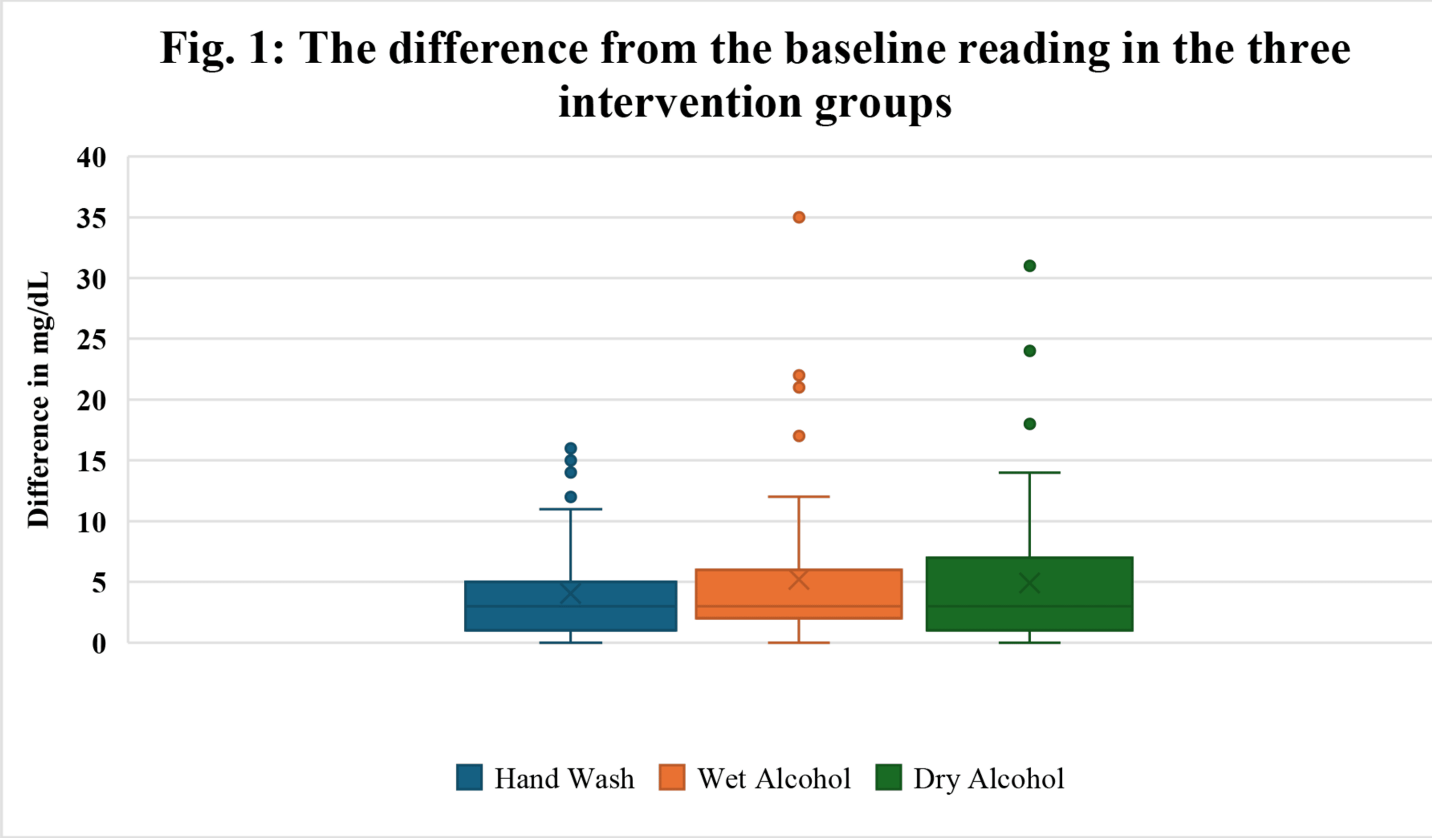
Box plot of the difference in blood glucose level (mg/dL) among different intervention groups from the baseline reading

Blood glucose levels were measured and compared across three intervention groups: hand washing, wet-alcohol swab, and dry-alcohol swab vs. baseline. The results of the Kruskal-Wallis test showed no statistically significant differences in glucose levels measured by the glucometer among the three interventions (p = 0.325) (Figure 1, Table 3).

**Table 3:** Comparison of Blood Glucose Levels (mg/dL) in the three Intervention Groups from the baseline reading *Kruskal–Wallis rank test The H statistic is 2.2488 (2, N = 249). The p-value is 0.32485. The result is not significant at p < .05. Calculation Summary: H = (12/(N(N+1)) * (∑T2/n) - 3(N+1), H = 0 * 3902290.645 – 750, H = 2.2488

Variable	Average difference from baseline reading	Maximum difference	Minimum difference	p-value
Hand wash	4.05	16	0	0.325^*^
Alcohol wet	5.23	35	0	
Alcohol dry	4.93	31	0	

## Discussion

This study assessed whether different finger-cleaning techniques influence the accuracy of blood glucose readings obtained using a glucometer. The comparison among hand washing with water, disinfection with 70% alcohol while still wet, and disinfection after complete drying revealed no statistically significant differences in blood glucose levels. Although the dry-alcohol group exhibited slightly higher readings, the difference was not clinically meaningful, suggesting that alcohol use prior to sampling does not substantially interfere with glucose measurement accuracy.

These findings align with previous studies conducted in Iran (2024) and Jeddah, Saudi Arabia (2022), which also reported no significant effect of alcohol swabbing on glucometer readings [12-14].

The results of this study hold important implications for clinical practice. In many healthcare settings, the use of alcohol swabs before finger-prick testing is a standard measure to prevent infection [7]. Some have expressed concerns that residual alcohol might alter glucose readings, leading to inaccurate results [8]. However, the present study demonstrates that both wet and dry alcohol disinfection methods are safe and do not significantly affect readings, thereby supporting continued adherence to disinfection protocols without compromising accuracy.

The study’s strengths include its quasi-experimental design and controlled measurement procedures. Nevertheless, several limitations should be acknowledged. First, the sample size of 249 participants, although adequate for preliminary analysis, may not have been sufficiently large to capture subtle variations across subgroups or to ensure strong statistical power. Future studies with larger and more diverse populations are recommended to confirm these findings and enhance generalizability. Second, all glucose measurements were obtained using a single glucometer brand and model, which may limit the generalizability of the findings to other devices with varying sensitivity or calibration methods. Additionally, instrument-related (technical) errors may have introduced minor measurement variability. Future research should explore the effects of alcohol disinfection across different glucometer brands and varying alcohol concentrations. Investigating these factors would further clarify whether specific conditions could amplify or minimize the minor differences observed in this study.

## Conclusions

The results of this study indicate that cleaning the fingertip with or without an alcohol swab before blood glucose measurement testing does not significantly affect the accuracy of glucometer readings, whether the alcohol is allowed to dry completely or not. Therefore, the use of alcohol swabs for hand disinfection remains a safe and practical practice in clinical and home glucose testing, provided that proper technique is followed.

## References

[1] Gregg EW, Sattar N, Ali MK (2016). The changing face of diabetes complications. Lancet Diabetes Endocrinol.

[2] (2025). Diabetes. https://www.who.int/health-topics/diabetes#tab=tab_1.

[3] Aljulifi MZ (2021). Prevalence and reasons of increased type 2 diabetes in Gulf Cooperation Council Countries. Saudi Med J.

[4] American Diabetes Association Professional Practice Committee (2024). 1. Improving care and promoting health in populations: standards of care in diabetes-2024. Diabetes Care.

[5] Klonoff DC (2014). Point-of-care blood glucose meter accuracy in the hospital setting. Diabetes Spectr.

[6] Jońca M, Krótki F, Tomasik P (2021). The effect of disinfecting procedure on the glucose concentration using a personal glucose meter. Prim Care Diabetes.

[7] (2025). WHO guidelines on drawing blood: best practices in phlebotomy. https://iris.who.int/items/5f1beec7-cb76-4699-a226-433c09de6145.

[8] (2025). Blood sugar testing: why, when and how. https://www.mayoclinic.org/diseases-conditions/diabetes/in-depth/blood-glucose/art-20046628.

[9] Sarah Vallie (2025). How to do a less painful finger prick. webmd.

[10] (2025). Blood collection procedure: capillary. https://pathlabs.ufl.edu/client-services/specimen-shipping/blood-collection-procedure-capillary/.

[11] (2025). Kingston Health Sciences Centre (KHSC). Facts about glucose monitoring. https://kingstonhsc.ca.

[12] Abdollahi M, Ayar A, Kouhpeikar H, Tavakol N, Hosseini SF (2024). Interfering effect of alcohol swabbing on capillary blood glucose concentration using a glucometer: a brief report. Mod Care J.

[13] Annisa N, Arwie D, Aryandi R (2024). Differences in the results of determining glucose levels using the POCT device on specimens taken when the finger is dry and on fingers that are still wet with 70% alcohol cotton. Front Sustain Sci Technol.

[14] Alzahrani A, Alshareef R, Binmahfoodh D (2022). Effect of hand hygiene practice on capillary blood glucose among the family medicine residents in Jeddah, Saudi Arabia. J Endocr Pract.

[15] Hortensius J, Slingerland RJ, Kleefstra N, Logtenberg SJ, Groenier KH, Houweling ST, Bilo HJ (2011). Self-monitoring of blood glucose: the use of the first or the second drop of blood. Diabetes Care.

[16] Stein C (2007). The effects of two differing techniques on the accuracy of reagent strip blood glucose testing. J Health SA Gesondheid.

[17] D'Orazio P, Burnett RW, Fogh-Andersen N (2005). Approved IFCC recommendation on reporting results for blood glucose (abbreviated). Clin Chem.

